# From Trauma to Recovery: A Comprehensive Management of Achilles Tendon Injury in a Young Female

**DOI:** 10.7759/cureus.52286

**Published:** 2024-01-15

**Authors:** Neha Arya, Anam Sasun, Ghanishtha Burile, Pallavi Harjpal, Rakesh K Kovela

**Affiliations:** 1 Neurophysiotherapy, Ravi Nair Physiotherapy College, Datta Meghe Institute of Higher Education and Research, Wardha, IND

**Keywords:** achilles tendon, case report, balance, strength, physiotherapy, rehabilitation

## Abstract

The incidence of the Achilles tendon getting injured has recently increased by 18 in 100,000. Compared to non-surgical treatment, surgical results are superior. The Achilles tendon repaired with surgery has a re-rupture rate of only 5%, while if treated non-operatively, it has a rupture rate of 40%. This case report analyses the traumatic Achilles tendon rupture and subsequent surgical repair in a young woman. In this case study, a 19-year-old female patient's severe Achilles tendon injury was successfully managed by integrating prompt surgical intervention and structure. After rehabilitation, the patient's range of motion (ROM), muscle strength, and gait patterns all significantly improved. Scores on the Lower Extremity Functional Scale (LEFS) and the Dynamic Gait Index (DGI) both significantly improved. This case study reiterates the significance of an integrated healthcare strategy for Achilles tendon injuries. An immediate surgical procedure followed by a specific rehabilitation programme accelerates healing and the return to optimal function. The results emphasize the critical role of physical therapy in assisting surgical interventions and underline the necessity of comprehensive patient care in the treatment of complex orthopaedic problems.

## Introduction

The Achilles tendon was first given that name in 1693 by Dutch physician Philip Verheyen in honour of the Greek hero Achilles [1]. The tendon is formed by two calf muscles, namely, the gastrocnemius and soleus, and is innervated by the sural nerve. The Achilles tendon is the biggest and strongest tendon in the human body [2]. The incidence of the Achilles tendon getting injured has recently increased by 18 in 100,000 [3]. Acute forceful plantar flexion of the foot, direct trauma, chronic tendinopathy, and intra-tendinous degenerative disorders can all result in Achilles tendon rupture [4,5]. Compared to non-surgical treatment, surgical results are superior. The Achilles tendon repaired with surgery has a re-rupture rate of only 5%, while if treated non-operatively, it has a rupture rate of 40%. The operative groups show high chances of developing infections and wound healing complications. Nowadays, due to advancing technologies, the incidences are decreasing. Lack of appropriate treatment facilities can cause lifelong complications. The tear is managed conservatively or non-conservatively. Physiotherapy intervention has been very beneficial [6,7].

It usually starts with a goal to decrease pain and swelling and subsequently work on regaining ankle movements, power, strength, and return to normal activities. Open repairs, minimally invasive procedures, and percutaneous approaches are all used to manage ruptured Achilles tendons. All of these procedures involve the insertion of sutures, which offer additional protection throughout the healing phase at the expense of cutting off the blood supply [8]. Achilles tendon rupture is mostly prevented by avoiding degenerative alterations within the tendon. Achilles tendon rupture prevention has also been connected to strengthening the ankle plantar flexors. Because of the force produced during eccentric contraction and with functional activity, strengthening of the eccentric plantar flexors is specifically advised [9,10]. When distal kinetic chain disturbances continue, the tendon's ability to absorb shock is reduced. This is similar to how increased external rotation of the lower leg begins with increased femoral anteversion to correct body alignment [11,12].

## Case presentation

Patient information

**Figure 1:**
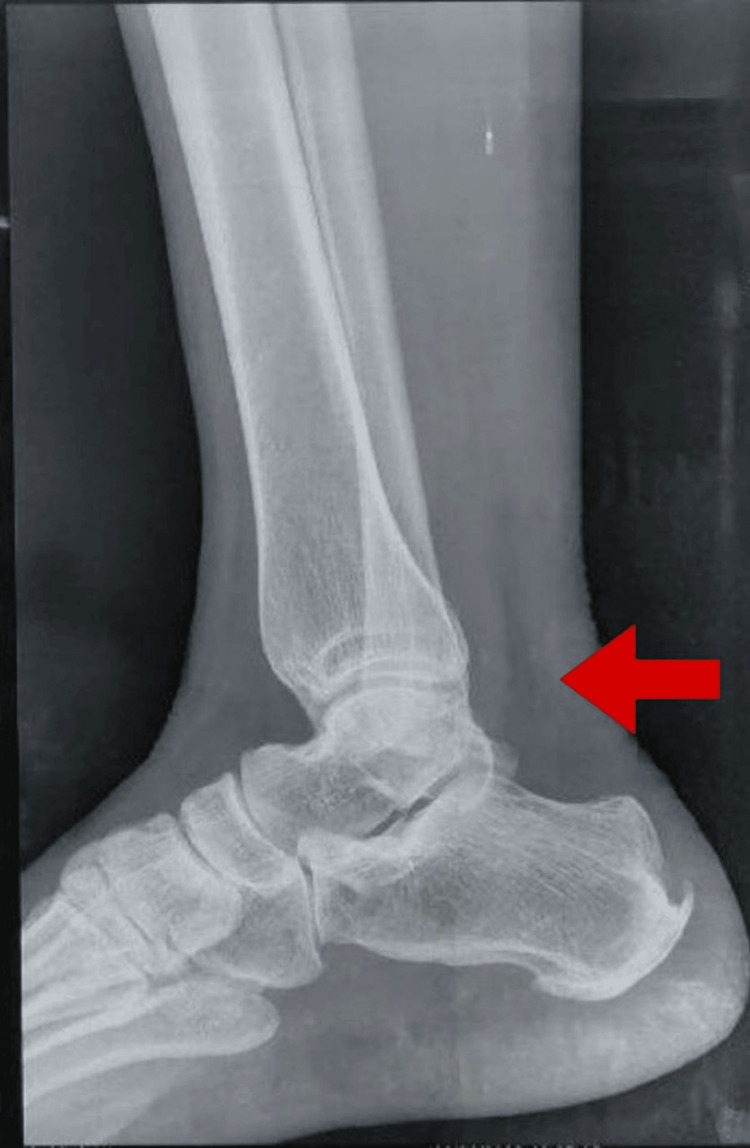
X-ray evidence of injury over the Achilles tendon (red arrow)

We are addressing the case of a 19-year-old female patient who worked as a daily wage labourer on a farm. She suffered from accidental trauma with a grass cutter machine at the back of her left foot, along with a pop-up sound and severe pain, swelling, and bleeding from the site of injury. She was immediately rushed to casualty in the nearby hospital. The attending doctor suspected it to be a torn Achilles tendon, along with a lacerated wound on her left ankle of approximately 6×4×3 cm. There was tenderness at the lateral malleolus, and ankle range of motion (ROM) couldn't be elicited due to injury. The consultant orthopaedic surgeon advised her of diagnostic procedures, i.e., X-ray. After the X-ray, the patient was referred for surgery. The patient was operated on in a prone lying position, the wound debridement was done and was nurtured with antiseptic, the tendon ends were repaired, and primary suturing of the wound was done. The repair was strengthened with Vicryl 1 suture (Ethicon Inc., Raritan, New Jersey, United States), and finally, the mending was completed following skin closure. The advantage of surgical repair is that the rate of rupture is very low. For three weeks, the wound was immobilized in a below-knee cast with 30° plantar flexion and non-weight bearing (NWB). On the third post-operative day, physical therapy was initiated. Removal of the cast was done 23 days after trauma, and progressive rehabilitation was planned for the patient. Figure 1 depicts an X-ray showing evidence of Achilles tendon injury.

Clinical assessment (after cast removal)

**Figure 2:**
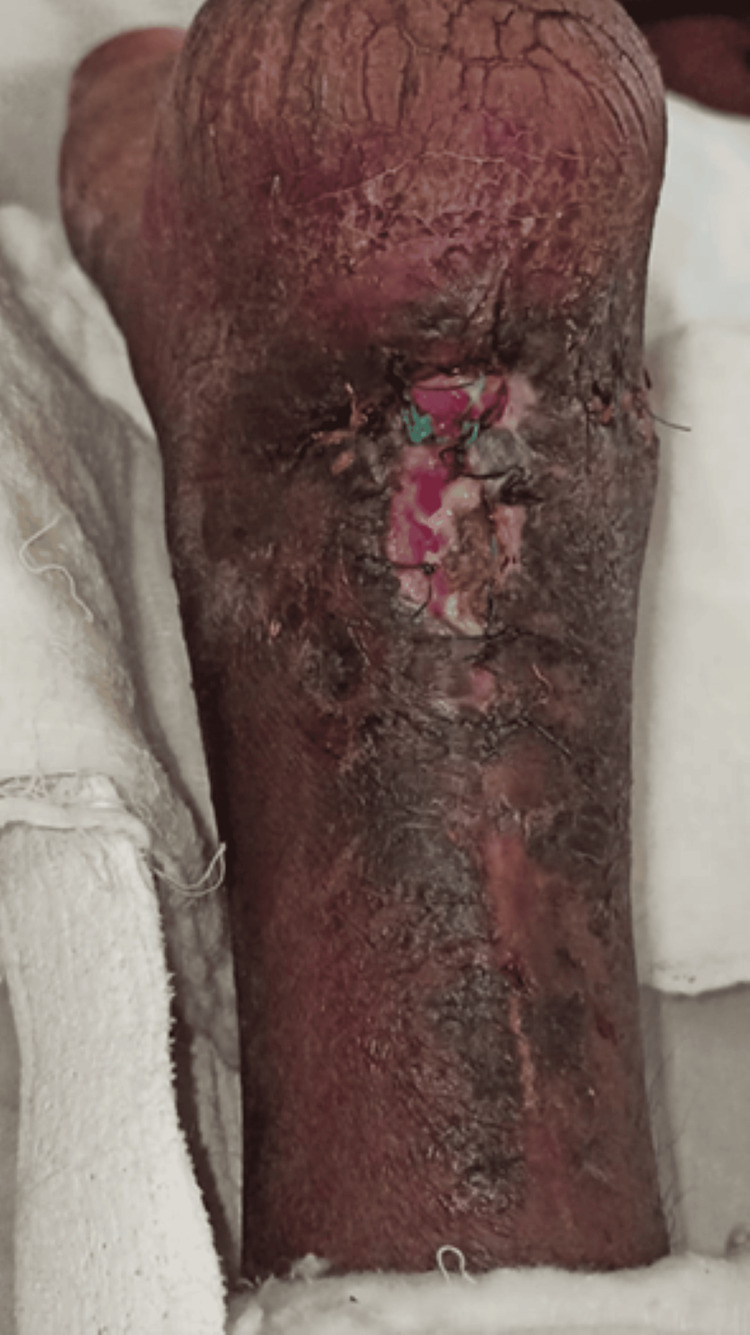
Injury site post surgery

Before beginning treatment, the patient gave their informed consent. On inspection, visible swelling was present near the ankle joint; a vertical scar mark was present on the posterior side of the ankle joint. Upon palpation, according to the Tenderness Grading Scale, there was Grade 1 tenderness, and the length of the scar was 4.7 cm. The patient complained of pain in her right ankle joint, which was described as dull aching with an intensity of 6/10 (on activity) and 4/10 (on rest) on the Numerical Pain Rating Scale. ROM and manual muscle testing examination were performed after the removal of the cast; there was a significant decrease in ankle ROM of the right leg, and dynamic gait index was taken for the assessment of balance and gait. The World Health Organization's Quality of Life Scale (WHO-QOL) was used to assess quality of life. In contrast, Barthel's index was used to assess dependence on activities of daily living (ADLs). Figure 2 shows the post-operative suture site.

Physiotherapy intervention

Physical therapy rehabilitation was planned for the patient. Treatment duration was seven weeks, six days per week. The protocol to be followed throughout the rehabilitation period is as follows: weeks 1-3 during plaster cast (NWB) and weeks 4-7 during full weight bearing. Table 1, Table 2, and Table 3 depict the physiotherapy protocol.

**Table 1:** Physiotherapy rehabilitation during weeks 1-3 (plaster cast) ROM: range of motion

Goals	Intervention	Repetitions
To maintain the mobility of hip and knee joint and prevent deep vein thrombosis	ROM exercises for the knee and hip; ankle toe movements (unaffected)	10 reps×1 set (2 times/day)
To maintain the strength of the quadriceps, hamstrings, and gluteal muscles	Isometrics: static quadriceps, static glutes, and static hamstrings	20 reps×1 set (2 times/day)
To maintain the strength of the unaffected limb	Strengthening exercises: dynamic quadriceps, hip abduction, and hamstring curls	10 reps×2 sets (2 times/day)

**Table 2:** Physiotherapy rehabilitation for weeks 4 and 5 (full weight bearing after cast removal) ROM: range of motion; MHz: megahertz; PNF: proprioceptive neuromuscular facilitation

Goals	Intervention	Repetitions
To reduce pain and promote the healing of the surgical site	Therapeutic ultrasound (week 4)	Duration: 8 mins. Mode: pulsed. Frequency: 1 MHz
To elongate the soft tissue	Slow dorsiflexion stretching (week 4)	3 reps×1 set (20-sec hold)
To improve ROM for ankle joint	Active assisted dorsiflexion, plantar flexion, inversion, eversion, and pedal exerciser (week 4)	10 reps×2 sets (2 times/day)
To improve the strength for ankle and foot muscles	Resistance band exercises for ankle and foot: yellow band (3 lbs) (week 5)	10 reps×1 set (2 times/day)
To improve the ROM and strength of ankle joint	PNF technique for ankle joint: contract-relax method (direct) (weeks 4 and 5)	5 reps×1 set (10-sec hold)
To improve balance and proprioception	Weight shifts in standing and tandem standing (week 5)	10 reps×1 set (2 times/day)
To improve gait pattern	Walking with the support of a walker (week 4); independent ambulation (week 5)	2-3 rounds (2 times/day)

**Table 3:** Physiotherapy rehabilitation for weeks 6 and 7

Goals	Intervention	Repetitions
To improve the strength of ankle and foot muscles	Resistance band exercises for the ankle and foot: red band (3.7 lbs) and green band (4.6 lbs). Ankle exerciser (weeks 6 and 7). Closed chain exercises: wall-supported squats and lunges (week 7)	10 reps×2 sets (2 times/day)
To improve balance and proprioception	Toe raises, heel raises, standing on one leg, spot marching, weight shifts on a Swiss ball, and wobble board balancing exercises (weeks 6 and 7)	10 reps×2 sets (2 times/day)
To improve gait pattern	Tandem walking, walking sideways, backwards walking, walking around obstacles, and walking over obstacles (weeks 6 and 7)	10 steps×2 sets (2 times/day)

**Figure 3:**
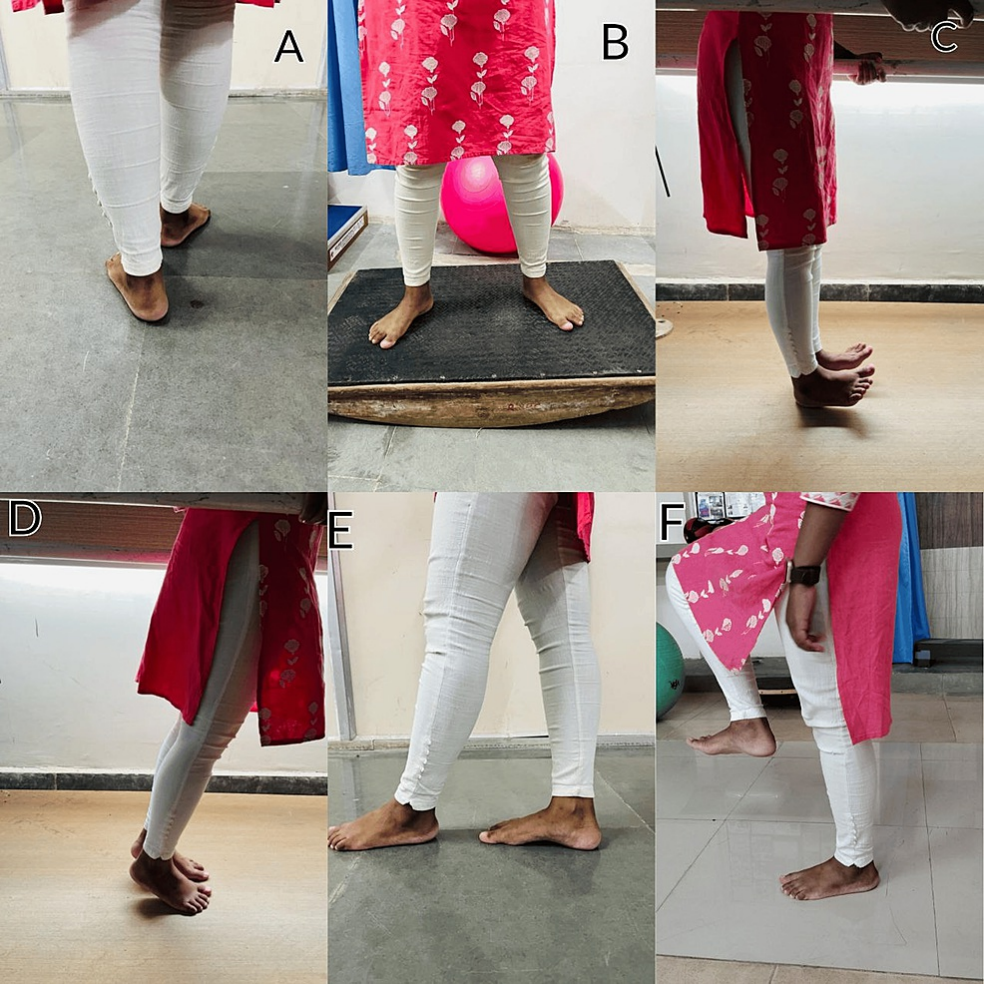
Balance and proprioception exercises A: tandem walking, B: wobble board exercise, C: heel raises, D: toe raises, E: tandem standing, F: spot marching

**Figure 4:**
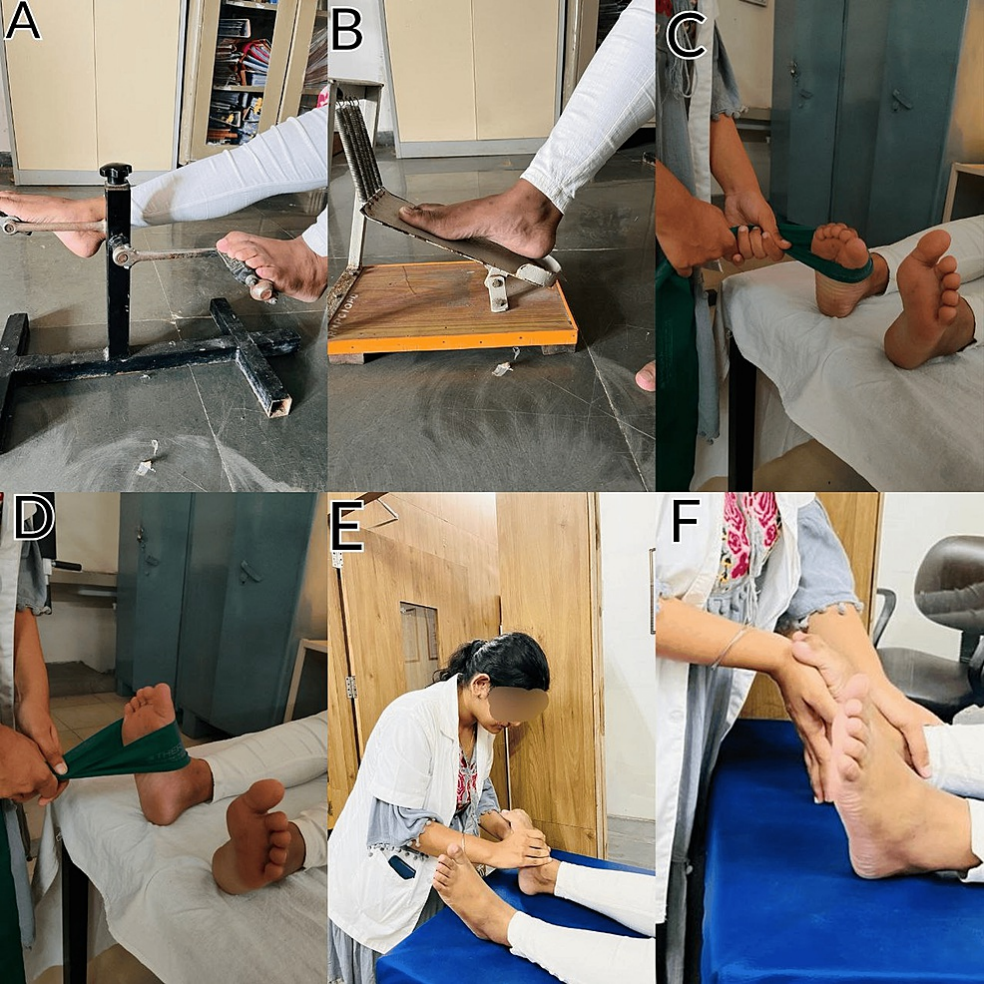
Strengthening, stretching, and ROM exercises A: pedal exerciser, B: ankle exerciser, C: plantar flexor strengthening by resistance band (green), D: dorsiflexor strengthening by resistance band (green), E: ankle PNF contract-relax method, F: Achilles tendon stretching ROM: range of movement; PNF: proprioceptive neuromuscular facilitation

Strengthening ROM exercises and balance and proprioception exercises are depicted in Figure 3 and Figure 4, respectively.

Follow-up and outcome measures

Table 4 and Table 5 shows the ROM and manual muscle testing findings pre and post rehabilitation. Table 6 depicts the outcome measures taken for the patient.

**Table 4:** Manual muscle testing (strength) for affected limb pre and post rehabilitation (Oxford grading) 3-: some but not complete ROM against gravity, 4: complete ROM against gravity with moderate resistance ROM: range of motion

Muscle group	Pre treatment	Post treatment
Dorsiflexors	3-	4
Plantar flexors	3-	4
Invertors	3-	4
Evertors	3-	4

**Table 5:** ROM assessment for the affected ankle joint (pre and post rehabilitation) ROM: range of motion

Movement	Pre treatment		Post treatment	
	Active	Passive	Active	Passive
Dorsiflexion	0-5°	0-10°	0-15°	0-18°
Plantar flexion	0-30°	0-35°	0-45°	0-50°
Inversion	0-10°	0-20°	0-25°	0-30°
Eversion	0-5°	0-5°	0-10°	0-15°

**Table 6:** Outcome measures WHO-QOL: World Health Organization's Quality of Life Scale

Outcome measure	Pre rehabilitation	Post rehabilitation
Numerical Pain Rating Scale	6/10	1/10
Lower Extremity Functional Scale	8/80	72/80
Dynamic Gait Index	4/24	23/24
WHO-QOL	40/100	82/100
Barthel's index	20/100	90/100

## Discussion

According to studies, 70% of the farmer population has a chance of getting such injuries due to negligence towards safety at work. The ruptures cause lifelong functional deficits. Individuals present with complaints even many years after injury. Nowadays, many studies are being carried out focusing on treatment strategies for tendon loading approaches. The benefit of control loading of tendons following rupture is very beneficial [13]. It has been demonstrated that early stress on structures, namely, tendons, improves mechanical properties [14], thereby improving functional outcomes. Due to delays in physiotherapy treatment, the patients might develop complications that can prolong their hospital stay and recovery process [15,16]. However, the differences observed in tendon metabolism and length were observed and reported in cases as well. The mechanical properties of the Achilles tendon improve gradually over time following ruptures. Early rehabilitation helps patients to return to basic living activities [17,18]. However, an increase in the amount of stress on ruptured structures is said to initiate re-modelling. It also provides insight into why re-rupture occurs in weaning immobilization. Early weight-bearing exercises should be given to decrease tendon elongation, improve the mechanical properties of tendons, and improve functional outcomes [19]. After radiological investigations, the patients are usually prescribed surgical treatment due to high-grade trauma [20].

In this case report, the patient underwent a structured physical therapy protocol supervised by a qualified physiotherapist. Rehabilitation commenced early, even while the cast was still applied. The primary objective during this phase was to preserve the inherent muscle strength. Once the cast was removed, a detailed evaluation was performed; this assessment revealed a noticeable reduction in the ROM of the affected ankle joint, a significant decrease in the strength of the ankle musculature, and impairments in both balance and gait. The Lower Extremity Functional Scale (LEFS) was used, indicating a compromised lower limb functionality, and the Dynamic Gait Index further underscored the balance and gait challenges. Responding to these findings, a tailored rehabilitation protocol was designed. This included strength training exercises using resistance bands, the integration of proprioceptive neuromuscular facilitation (PNF) techniques, targeted exercises for balance and proprioception, and activities intended to restore regular walking patterns. Following a dedicated seven-week rehabilitation period, the patient demonstrated marked improvements in ROM, ankle musculature strength, lower limb function as measured by LEFS, and balance and gait function; following rehabilitation, there was also a noticeable improvement in independence for ADLs and quality of life, as measured by Barthel's index and WHO-QOL.

## Conclusions

This case report underscores the critical importance of timely and integrated care for Achilles tendon injuries. The patient's swift progression from trauma to recovery illustrates the profound impact of immediate surgical intervention paired with structured physiotherapy. It's evident that a holistic, interdisciplinary approach, combining precise diagnostics, surgery, and tailored rehabilitation, is key to optimizing outcomes and restoring function swiftly. This underscores the significance of collaborative healthcare in tackling intricate orthopaedic challenges; the study concludes that the help of appropriate surgical intervention along with early and effective physical rehabilitation protocol leads to enhancement in the functional and mobility goals of the patient with an Achilles tendon injury.
